# Sublethal Oxidative Stress Induces the Premature Senescence of Human Mesenchymal Stem Cells Derived from Endometrium

**DOI:** 10.1155/2013/474931

**Published:** 2013-08-25

**Authors:** Elena Burova, Aleksandra Borodkina, Alla Shatrova, Nikolay Nikolsky

**Affiliations:** Department of Intracellular Signaling and Transport, Institute of Cytology of Russian Academy of Sciences, St. Petersburg 194064, Russia

## Abstract

The specific responses of mesenchymal stem cells to oxidative stress may play a crucial role in regulation of tissue homeostasis as well as regeneration of organs after oxidative injury. The responses of human endometrium-derived mesenchymal stem cells (hMESCs) to oxidative stress remain still unknown. Herein, we examined the impact of H_2_O_2_ on cell viability, induction of premature senescence, and apoptosis. hMESCs were highly resistant to H_2_O_2_ compared with human diploid fibroblasts. To test a hypothesis whether hMESCs may undergo oxidative stress-induced premature senescence, cells were briefly exposed to the sublethal H_2_O_2_ doses. H_2_O_2_-treated cells were permanently arrested, lost Ki67 proliferation marker, and exhibited a senescent phenotype including cell hypertrophy and increased SA-**β**-Gal activity. Additionally, in stressed cells the expression levels of p21Cip1, SOD1, SOD2, and GPX1 were elevated. hMESCs survived under stress were not able to resume proliferation, indicating the irreversible loss of proliferative potential. While the low H_2_O_2_ doses promoted senescence in hMESCs, the higher H_2_O_2_ doses induced also apoptosis in a part of the cell population. Of note, senescent hMESCs exhibited high resistance to apoptosis. Thus, we have demonstrated for the first time that hMESCs may enter a state of premature senescence in response to sublethal oxidative stress.

## 1. Introduction

Stress responses of human embryonic and adult stem cells to *γ*-radiation, oxidative stress, heat shock, and so forth are widely researched to establish cell-based strategies of tissue repair, tissue engineering, and transplantation [1]. Human mesenchymal stem cells are adult multipotent stem cells with the capacity of self-renewal and undergoing adipogenic, osteogenic, chondrogenic, myogenic differentiation [2, 3]. They contribute to the homeostatic maintenance of many organs and tissues [4, 5]. Unlike some other adult stem cells (e.g., hematopoietic stem cells) human mesenchymal stem cells are not immortal. These cells exhibit ex vivo growth characteristics typical of the Hayflick model of cellular senescence with a limited life span [6]. Recently, it has been reported that the mesenchymal stem cells subjected to oxidative stress [7–9] or ionizing radiation [10–12] may undergo stress-induced premature senescence in vitro. Many types of normal and tumor cells also enter a state of premature senescence after exposure to radiation [13–15], H_2_O_2_ [16–19], or treatment with histone deacetylase inhibitors [20, 21]. Prematurely senescent cells exhibit some of the characteristics inherent in replicatively senescent cells, including a large flat morphology, increased senescence-associated *β*-galactosidase (SA-*β*-Gal) activity, and permanent cell cycle arrest [14, 17]. Besides, cellular overactivation and hyperfunction, feedback signal resistance, and loss of regenerative potential are considered hallmarks of senescence [22]. Progress in understanding the causes and mechanisms of cellular senescence and significance of senescence for ageing and suppressing cancer has been reviewed [23–25].

In the current study, oxidative stress responses of human mesenchymal stem cells derived from endometrium (hMESCs) were investigated. Our knowledge of specific responses of these cells to stress is very limited, though they prove to be useful in the treatment of pathologies in which tissue damage is linked to oxidative stress. Unlike most of the human mesenchymal stem cells, the isolation of which as a rule is complicated by invasive procedures, the mesenchymal stem cells produced from desquamated endometrium in menstrual blood by a simple noninvasive way provide a good opportunity to explore the stress responses of hMESCs. Regarding hMESCs, phenomenon of premature senescence induced by oxidative stress remains still unknown. This study aimed to test a hypothesis whether hMESCs after exposure with sublethal doses of H_2_O_2_ may undergo the stress-induced premature senescence. In parallel, the impact of H_2_O_2_ on cell viability and development of apoptosis has been evaluated.

## 2. Materials and Methods

### 2.1. Cell Culture and Cell Treatment

Human mesenchymal stem cells isolated from desquamated endometrium in menstrual blood (hMESCs, line 2304), as described previously [26], as well as human embryonic lung-derived diploid fibroblasts (HDF, line FRL-9505) were cultured in complete medium (DMEM/F12 (Gibco BRL, Gaithersburg, MD, USA) supplemented with 10% FBS (HyClone, Waltham, MA, USA), 1% gentamycin, and 1% glutamax (Gibco BRL, Gaithersburg, MD, USA)) at 37°C in humidified incubator, containing 5% CO_2_. hMESCs have a positive expression of CD73, CD90, CD105, CD13, CD29, and CD44 markers and absence of expression of the hematopoietic cell surface antigens CD19, CD34, CD45, CD117, CD130, and HLA-DR (class II). Multipotency of isolated hMESCs is confirmed by their ability to differentiate into other mesodermal cell types, such as osteocytes and adipocytes. Besides, the isolated hMESCs partially (over 50%) express the pluripotency marker SSEA-4 but do not express Oct-4. Immunofluorescent analysis of the derived cells revealed the expression of the neural precursor markers nestin and beta-III-tubulin. This suggests a neural predisposition of the established hMESCs. These cells are characterized by high rate of cell proliferation (doubling time 22-23 h) and high cloning efficiency (about 60%). Cells at early passages (between 6 and 9 passages for hMESCs and between 16 and 21 passages for HDF) were used in all experiments to avoid complications of replicative senescence. Cells were harvested by trypsinization and plated at a density of 15 × 10^3^ cells per cm^2^. For microscopy experiments, cells were grown on glass coverslips. H_2_O_2_ treatments were performed on subconfluent cells to avoid variability of H_2_O_2_ toxicity. H_2_O_2_ stock solution in serum-free medium was prepared from 30% H_2_O_2_ (Sigma, St. Louis, MO, USA) just before adding. Cells were treated with H_2_O_2_ in the range of concentrations from 200 *μ*M to 2,000 *μ*M for 1 h for MTT assay. Based on LD_50_ values 200 *μ*M H_2_O_2_ was chosen as a sublethal concentration for the induction of premature senescence of hMESCs, whereas apoptosis was tested under higher concentrations of H_2_O_2_ (900 and 3,000 *μ*M). The cells were washed twice with serum-free medium to remove H_2_O_2_ and then recultured in fresh complete medium for various durations as specified in individual experiments.

### 2.2. Assessment of Cell Viability

The cell viability after exposure to H_2_O_2_ for 1 h was evaluated by the enzymatic conversion of MTT (AppliChem, Darmstadt, Germany, number A2231) to formazan in live cells. The culture medium from the cells grown in plates was removed, and 3-(4.5-dimethylthiazol-2-yl)-2.5-diphenyltetrazolium bromide (MTT; 0,715 mg/mL) in serum-containing growth medium was added to each well. In 2 h the solution was changed to DMSO to solve formazan produced. The plates were shaken for 15 min at room temperature; thereafter the absorbance was measured at 570 nm using microplate reader (Fluorofot “Charity,” Russia). All points were read as parallels of 8 similar samples. The average absorbance at a given time point was normalized to the start time point.

### 2.3. Flow Cytometry

Adherent cells were rinsed twice with PBS and harvested by trypsinization. Detached cells were collected with supernatants, pelleted by centrifugation. Detached and adherent cells were finally pooled and resuspended in PBS. One part of each sample was used for propidium iodide (PI) staining to evaluate cell viability and another part for cell cycle phase distribution analysis that was performed as described previously [27]. 50 *μ*g/mL PI was added to each sample just before analysis and mixed gently. Samples were analyzed on a Coulter EPICS XL Flow Cytometer (Backman Coulter, Brea, CA, USA). For cell cycle analysis, each cell sample was suspended in 300 *μ*L PBS containing 200 *μ*g/mL of saponin (Fluka, NY, USA), used for cell permeabilization, 250 *μ*g/mL RNase A (Sigma, St. Louis, MO, USA, number R4642), and 50 *μ*g/mL PI, incubated for 30 min at room temperature and subjected to FACS analysis. At least 10,000 cells were measured per sample. Cell cycle analysis was performed using Win MDI program version 2.8 and ModFit LT software (Verity Software House, Topsham, ME, USA).

### 2.4. FACS Analysis of Cell Enlargement

The same procedure of sample preparation as described previously was done for light-scattering cytometry. As after H_2_O_2_ treatment live and dead cells were very close to each other on one-parameter histogram, two-parameter histogram was used (FL4LOG versus FSLOG) to discriminate live and dead cells. Analysis of each sample was performed for 100 sec with high sample delivery. Cell size of control and H_2_O_2_-treated cells was measured by means of cytometric light scattering of PI-stained cells by using Win MDI program version 2.8.

### 2.5. SA-*β*-Gal Activity

Cells expressing senescent-associated *β*-galactosidase were detected with senescence *β*-galactosidase staining kit (Cell Signaling Technology, Beverly, MA, USA, number 9860) according to manufacturer's instructions. The kit detects *β*-galactosidase activity at pH 6 in cultured cells which is present only in senescent cells and is not found in presenescent, quiescent, or immortal cells. The percent of SA-*β*-Gal-positive cells was calculated by counting not less than 500 cells.

### 2.6. Immunofluorescence Staining

Cells cultured on coverslips were fixed with PBS/4% formalin for 15 min and then permeabilized with 0.1% Triton X-100. After blocking with 1% bovine serum albumin, they were incubated with a rabbit polyclonal antibody against Ki67 (Abcam, Cambridge, UK, number 15580) (1 : 1000) overnight at 4°C and then with Alexa Fluor 568 donkey anti-rabbit antibody (Invitrogen, Carlsbad, USA, number A10042) (1 : 500) at room temperature for 1 h after extensive washing with PBS/0.1% Tween 20 between each step. The slides were counterstained with 1 *μ*g/mL DAPI (Sigma, St. Louis, MO, USA, number D9564) and mounted using 2% propyl gallate. A Zeiss Axiovert 200M fluorescence microscope (Carl Zeiss, Germany) equipped with a digital camera DFC 420C (Leica, Germany) utilizing Adobe Photoshop software was used to view and acquire images.

### 2.7. Apoptosis Detection

Apoptosis detection was performed by Annexin V/PI staining according to standard manufacture protocols (BD Pharmingen). H_2_O_2_-treated and control cells were harvested as described previously. Then cells were washed twice with cold PBS, resuspended in 1X binding buffer at concentration 10^6^ cells/mL. 100 *μ*L of this suspension was transferred to 5 mL tube, and 5 *μ*L Annexin V/FITC (Annexin V/FITC Apoptosis Detection Kit II, BD Biosciences, San Diego, CA, USA) and 10 *μ*L PI were added; suspension was gently mixed and incubated for 15 min at room temperature in the dark. Just before flow cytometric analysis 400 *μ*L of 1X binding buffer was added to each sample.

### 2.8. Western Blotting

Total cell lysates were prepared as described previously [27]. Protein content was determined by the method of Bradford. The cell lysates were dissolved in SDS sample buffer and separated on 8% or 12% SDS gel. SDS-PAGE electrophoresis, transfer to nitrocellulose membrane, and immunoblotting with ECL (Thermo Scientific, USA) detection were performed according to standard manufacturer's protocols (Bio-Rad Laboratories, USA). The following antibodies were used: rabbit monoclonal antibodies against *p*21^Waf1/Cip1^ (12D1, number 2947S) (1 : 1000) and against glyceraldehyde-3-phosphate dehydrogenase (GAPDH, clone 14C10, number 2118S) (1 : 1000), as well as horseradish peroxidase-conjugated goat anti-rabbit IG (number 7074S) (1 : 10000). All antibodies were purchased from Cell Signaling, USA. Hyperfilm (CEA) was from Amersham (Sweden). Equal protein loading was confirmed by Ponceau S (Sigma, St. Louis, MO, USA, number P7170) staining.

### 2.9. RT-PCR Assay

To analyze gene expression, total RNA from cells was isolated with RNesy Micro Kit (Qiagen, USA) according to manufacturer's instructions. cDNA synthesis was performed with 1 *μ*g of total RNA using RevertAid H Minus First Strand cDNA Synthesis Kit (Fermentas, Lithuania) according to manufacturer's instructions. Specific genes were amplified by Taq DNA polymerase (Fermentas, Lithuania) with C1000 Touch Thermal Cycler amplifier (Bio-Rad Laboratories, USA). The program was as follows: hot start-denaturation at 93°C for 3 min, primer annealing at 51–70°C for 2 min, and then elongation at 72°C for 1 min 30 sec; thereafter, 25–27 cycles of denaturation at 93°C for 45 sec, primer annealing at 51–70°C for 1 min, elongation at 72°C for 1 min 30 sec, and then final elongation at 72°C for 10 min; primers *p*21^*Waf*1/*Cip*1^, sense 5′ CCA CAT GGT CTT CCT CTG CTG 3′, antisense 5′ GAT GTC CGT CAG AAC CCA TG 3′, annealing temperature 55°C (316 bp); *SOD1*, sense 5′ GGT CCT CAC TTT AAT CCT CTA T 3′, antisense 5′ CAT CTT TGT CAG CAG TCA CAT T 3′, annealing temperature 62°C (96 bp); *SOD2*, sense 5′ TGA CAA GTT TAA GGA GAA GC 3′, antisense 5′ GAA TAA GGC CTG TTG TTC C 3′, annealing temperature 56°C (148 bp); *GPX1*, sense 5′ CGC CAC CGC GCT TAT GAC CG 3′, antisense 5′ GCA GCA CTG CAA CTG CCA AGC AG 3′, annealing temperature 68°C (238 bp); *β*-*actin* gene was used for RNA quantitative control and DNA contamination monitoring: sense primer 5′-GCC GAG CGG GAA ATC GTG CGT-3′, antisense 5′-CGG TGG ACG ATG GAG GGG CCG-3′, annealing temperature 70°C (507 bp). All primers were obtained from SYNTOL (Russia). The electrophoresis of amplified products was performed in 2% agarose gel with TAE buffer and ethidium bromide. 100 kb DNA ladder (Fermentas, Lithuania) was used as molecular weight markers. Amplified products were visualized in UV light (302 nm) with transilluminator and registered with a digital Canon camera.

### 2.10. Statistics

All data are presented as the mean and standard error of the mean from at least three separate experiments performed. Statistical differences were calculated using the Student's *t*-test and considered significant at *P < 0.05.

## 3. Results and Discussion

### 3.1. Cell Viability under Oxidative Stress

**Figure 1:**
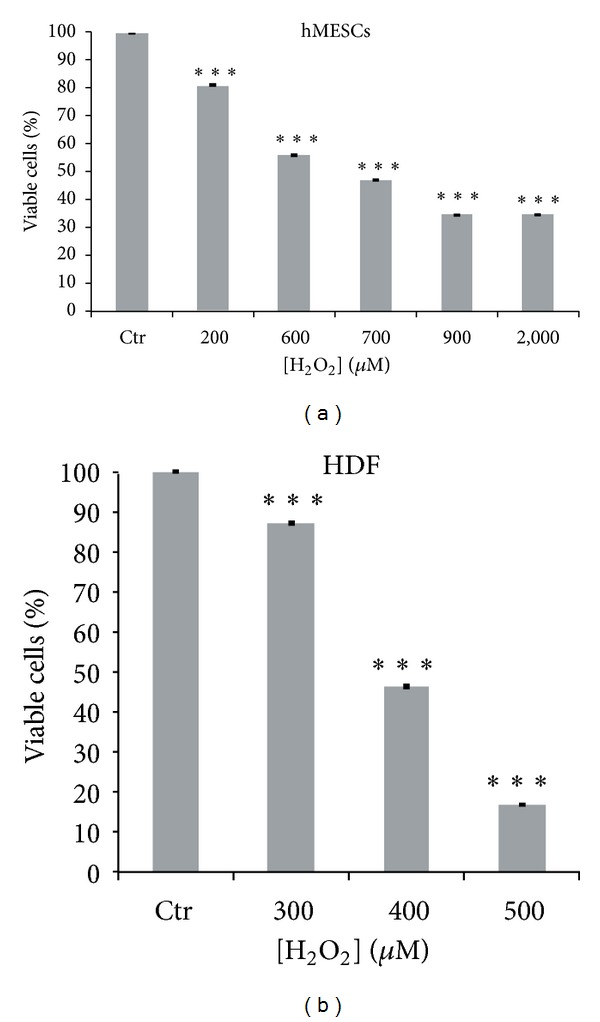
The viability of hMESCs and HDF under oxidative stress. Cells were either treated or not with H_2_O_2_ at indicated concentrations for 1 h. The percentage of viable cells was evaluated in 24 h after treatment using MTT assay as described in Section 2. Results are shown as a percent of control. Data represent mean ± SEM of at least three independent experiments. ***P < 0.001 significantly different from the untreated control cells. LD values were 600–700 and 370–400 *μ*M for hMESCs and HDF, respectively. Control (Ctr): untreated cells.

H_2_O_2_ treatment of cultured cells is a commonly used model to test oxidative stress susceptibility in different cell types. Accumulating evidence pointed to a high resistance of mesenchymal stem cells to oxidative stress caused by H_2_O_2_ [28, 29]. On the other hand, it has been recently reported that some types of human mesenchymal stem cells are very sensitive to H_2_O_2_ exposure [9]. Susceptibility of hMESCs to oxidative stress remains unexplored up to date. We earlier reported that hMESCs subjected to prolonged treatment (for 24 h) with H_2_O_2_ demonstrated a higher resistance compared with human diploid fibroblasts [30]. In this study, a pulse cell treatment with H_2_O_2_ in varying concentrations from 200 *μ*M to 2 mM for 1 h was applied. Human diploid fibroblasts (HDF) were used as a H_2_O_2_ sensitive cell model to compare effect of H_2_O_2_ cytotoxicity on hMESCs. Firstly, it was necessary to assess hMESCs viability under oxidative stress to examine a sublethal H_2_O_2_ concentration required for further experiments. Previously we have found out that, at a fixed H_2_O_2_ concentration, both cytotoxicity and rate of degradation were dependent on the volume of H_2_O_2_ solution added to the culture medium; therefore the volume used was adjusted proportionally according to the surface area in order to obtain a consistent H_2_O_2_ cytotoxicity. In addition, it was very important to control plating cell density because the H_2_O_2_ effect (at equal volume and concentration) on cells was inversely related to cell density; that is, confluent cell cultures were more resistant to H_2_O_2_ than subconfluent ones. Cell viability was evaluated by MTT assay as a broad indicator of cellular activity that allows estimating a number of viable cells via monitoring mitochondrial dehydrogenase activity. As shown in Figure 1, H_2_O_2_ affected the cell viability of both cell lines in a dose-dependent manner. LD_50_ values characterizing cell viability corresponded to 600–700 *μ*M (15–17.5 pmol/cell) for hMESCs and 370–400 *μ*M (3.5–4.0 pmol/cell) for HDF. Thus, hMESCs were found to be very resistant to H_2_O_2_ compared to HDF.

### 3.2. Expression of Antioxidant Enzymes

**Figure 2:**
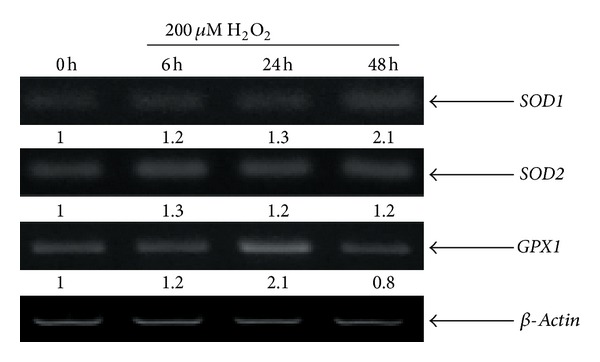
Gene expression of *SOD1*, *SOD2,* and *GPX1* is enhanced in hMESCs in response to H_2_O_2_ treatment. Exponentially growing cells were treated with the sublethal dose (200 *μ*M) of H_2_O_2_ for 1 h with following H_2_O_2_ replacement and then cultured under normal conditions for the indicated time. Total RNA isolated from hMESCs was amplified with specific primers for referred genes. *β-Actin* was used as a loading control.

To examine whether this high resistance to effect of H_2_O_2_ correlates with the ability of hMESCs to effectively scavenge reactive oxygen species (ROS), expression of the genes coding for enzymes involved in the elimination of ROS such as SOD1, SOD2, and GPX1 was tested. Previously, we have observed a rapid increase of the intracellular ROS levels in hMESCs after exposure to H_2_O_2_ (data not presented). Here, it was shown that, in untreated cells, the basal levels of mRNA expression of all of the antioxidant enzymes were elevated, while after treatment with 200 *μ*M H_2_O_2_ an expression level of each enzyme was upregulated to different extent (Figure 2). Consequently, the significant insensitivity to H_2_O_2_ was consistent with the enhanced expression levels of the antioxidant enzymes. These findings are in agreement with previous report demonstrating a high resistance of human bone marrow-derived mesenchymal stem cells to oxidative stress [29]. The conflicting findings demonstrating a particular sensitivity of human umbilical cord blood-derived mesenchymal stem cells to H_2_O_2_ have correlated with the low levels of antioxidant enzyme activity [9].

### 3.3. A Sublethal Oxidative Stress Induces a Premature Senescence Phenotype in hMESCs

**Figure 3:**
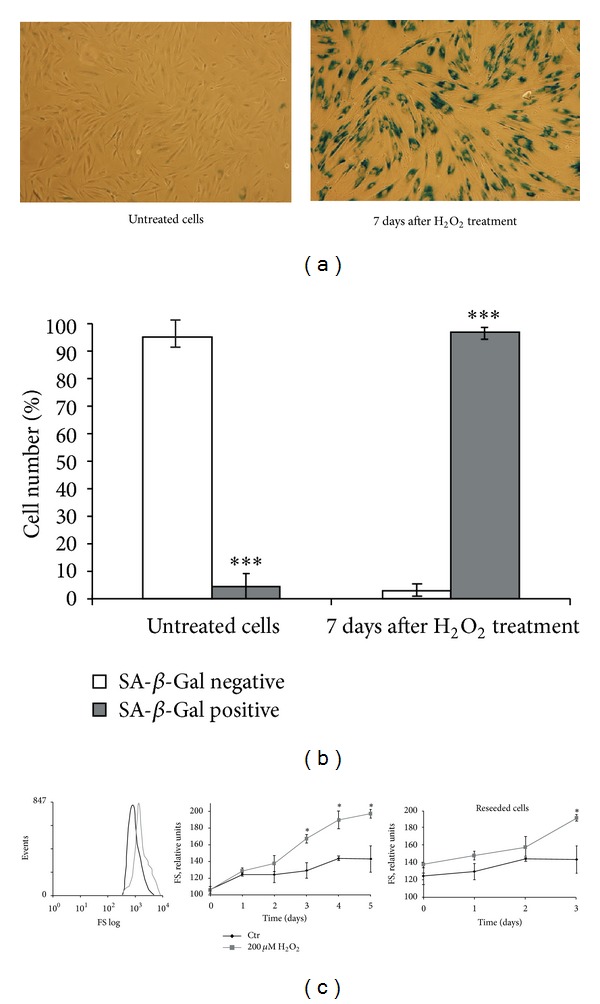
The sublethal dose of H_2_O_2_ induces senescent phenotype in hMESCs. Cells were treated as indicated in the legend of Figure 2. (a) SA-*β*-Gal staining. (b) Quantitative assay of SA-*β*-Gal-positive cells. (c) H_2_O_2_-induced cell size increase. Typical presentation of forward scatter (FS), reflecting the average cell size (left). Cell size was determined daily: H_2_O_2_-treated cells were either cultured for 5 days under standard conditions (middle) or were reseeded in 2 days and additionally cultivated for 3 days (right). Data were obtained by light-scattering cytometry with using Win MDI program version 2.8. Data represent mean ± SEM of at least three independent experiments. Significant difference was based on the Student's *t*-test (*P < 0.05, ***P < 0.001). Control (Ctr): untreated cells.

In hMESCs, phenomenon of H_2_O_2_-induced premature senescence was not so far described. In all experiments, early-passage cells were used to avoid undesirable replicative senescence of cells, because the major features of both replicative and stress-induced senescence are known to be alike [14, 17, 18]. To test whether hMESCs after treatment with a sublethal H_2_O_2_ concentration (200 *μ*M) could undergo SIPS, we assessed a variety of senescent-associated biomarkers: change of cell morphology, SA-*β*-Gal staining, increasing of cell size, loss of proliferative potential, cell cycle arrest, and p21^Waf1/Cip1^ (hereafter p21) status. H_2_O_2_ treatment was found to lead to development of senescent-like morphology: cells become enlarged, flattened, and heterogeneous. It should be noted that, in a part of cell population, we could see such morphological changes within 24 h after H_2_O_2_ treatment. In addition, senescent cells demonstrated SA-*β*-Gal staining which increased gradually, and the most remarkable effect was reached at 7 days after treatment: more 95% H_2_O_2_-treated cells were SA-*β*-Gal positive (Figures 3(a) and 3(b)). Exponentially growing control cells displayed very weak, if any, increase of SA-*β*-Gal staining. Importantly, a premature senescence phenotype was maintained during the follow-up period of 21 days (data not shown).

The increased heterogeneity of the cellular size of H_2_O_2_-treated hMESCs was further confirmed and quantified by light-scattering cytometry of PI-stained cells. As indicated in Figure 3(c), H_2_O_2_ induced a 2-fold increase of cell size after 5 days compared with control, as measured by the shift in the mean value of the forward scatter. The elevated size of treated cells was sustained constant, at least, for 5 days, whereas the size of control cells was almost not changed. To test whether treated hMESCs retain their increased size being reseeded, after H_2_O_2_ treatment cells were cultured for 2 days and then reseeded and additionally cultured under normal cell culture conditions for 3 days. As a result, we observed the similar increase of cell size (1.8-fold) in both reseeded and cultured cells for 5 days without reseeding cells (Figure 3(c)). Importantly, cell size increase was accompanied with protein content elevation, suggesting protein synthesis in H_2_O_2_-treated cells. Together, the results obtained demonstrate cellular hypertrophy within hMESCs population in response to H_2_O_2_. 

### 3.4. The Permanent Cell Cycle Arrest and Loss of the Proliferative Potential in hMESCs Subjected to Sublethal Oxidative Stress

**Figure 4:**
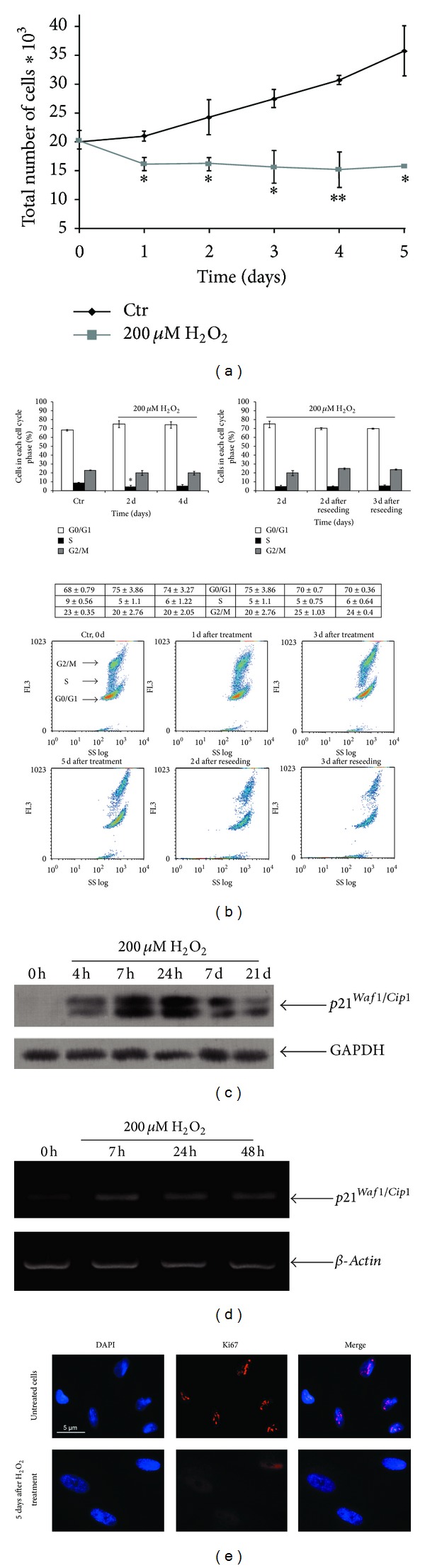
Induction of the premature senescence in hMESCs under oxidative stress leads to the permanent arrest of cell cycle and irreversible loss of proliferative potential. Cell treatment was done as described in Figure 2. (a) Growth curve of both H_2_O_2_-treated and untreated cells. Cell number was determined daily after cell exposure to H_2_O_2_ by FACS analysis (M ± SEM, *n* = 3, *P < 0.05, **P < 0.01). (b) H_2_O_2_-treated cells were either cultured for 5 days or were reseeded in 2 days and additionally cultured for 3 days. Flow cytometry analysis of cell cycle phase distribution: the percentage of cells in the G0/G1, S, and G2/M phases (upper panel) (*P < 0.05); visualization of phase distribution based on light-scattering analysis (lower panel); SS: side scattering, FL3: PI fluorescence. (c) The expression levels of p21 protein. Representative results of the three experiments are shown in the figure. (d) The levels of *p21* mRNA expression. GAPDH and *β-actin* were used as loading controls. (e) The nuclear localization of Ki67 was tested in control or H_2_O_2_-treated cells by immunofluorescence and DAPI staining. Representative photomicrographs of the staining are shown. Images were taken at magnification 100x. Control (Ctr): untreated cells.

In order to further characterize H_2_O_2_-induced senescent-like state of hMESCs, we analyzed their proliferative potential. Cell number in both untreated and H_2_O_2_-treated cell cultures was counted during 5 days. As seen in Figure 4(a), the pattern of growth curves indicates a significant increase (more than two times in 5 days) in the number of proliferating control cells compared with H_2_O_2_-treated cells. Consequently, 200 *μ*M H_2_O_2_ caused a permanent growth arrest, that is, a permanent loss of the proliferative potential. Additionally, a proliferative status of cells was examined by staining with antibodies against proliferation marker Ki67. As seen in Figure 4(e), in 5 days after H_2_O_2_ treatment, there were no Ki67-positive cells in the cell culture, while the proliferating control cells had a pronounced staining. As viability of hMESCs in response to 200 *μ*M H_2_O_2_ did not decrease appreciably (Figure 1), we suggested that H_2_O_2_-induced growth inhibition of hMESCs could be associated with rather the cell cycle arrest than promotion of cell death.

The analysis of the cell cycle phase distribution in hMESCs showed that a pulse H_2_O_2_ treatment led to the arrest in all of the cycle phases (Figure 4(b), upper panel). Treated cells demonstrated the prolonged arrest, at least, for 5 days. The phase distribution of treated cells in each time point tested was characterized with a minor accumulation of cells in G0/G1 phase compared with control cells. The distribution analysis with using light scattering confirmed these findings (Figure 4(b), lower panel). To test whether arrested cells could recover their proliferative potentials, in 2 days after treatment cells were reseeded and cultivated for 3 more days. As expected, reseeded cells also displayed the cell cycle arrest. Moreover, cell cycle phase distributions of both reseeded and taken-before-reseeding cells were identical (Figure 4(b)). Consequently, the senescent cells were not able to resume proliferation even after being reseeded, indicating the irreversible growth arrest. These observations were confirmed by proliferation assay (Figure 4(a)), which demonstrated that H_2_O_2_-treated cells were not able to proliferate normally for 5 days. Overall, these results suggest that cellular senescence in hMESCs was induced through growth arrest by H_2_O_2_. 

### 3.5. A Permanent Loss of the Proliferative Potential in hMESCs Is Accompanied with Elevated Levels of p21

In human mesenchymal stem cells, cyclin-dependent kinase inhibitor p21 was recently shown to be upregulated during H_2_O_2_-induced premature senescence [7, 8]. Increased levels of p21 may mediate the initiation of H_2_O_2_-induced cell cycle arrest by inhibiting various cyclin-dependent kinases that contribute cell cycle phase progression [14, 16]. To find out whether p21 could be involved in the regulation of H_2_O_2_-induced senescence of hMESCs, protein and mRNA expression levels of p21 were determined. 200 *μ*M H_2_O_2_ promoted a significant elevation in protein (Figure 4(c)) and mRNA (Figure 4(d)) expression of p21 in 7 h after treatment. An inducible expression of p21 was upregulated during 1-2 days with a following decline to insignificant, but not control, levels and was accompanied with the cell cycle arrest at the same time (Figure 4(b)). Importantly, the arrested cells thereafter could acquire a senescent morphology (Figure 3(a)) but could not resume proliferation (Figure 4(a)). We assume that the elevated p21 expression is essential to drive H_2_O_2_-induced premature senescence in hMESCs. In support of our findings, it has been reported that, in bone marrow-derived mesenchymal stem cells exposed to sublethal doses of H_2_O_2_, a rapid decrease of proliferation rate was detected within 3 days and correlated with G1 phase arrest of the cell cycle when p21 was accumulated at the same time [8].

In summary, our findings strongly indicate that hMESCs under a sublethal oxidative stress are able to undergo premature senescence.

### 3.6. Effect of High H_2_O_2_ Doses on hMESCs

**Figure 5:**
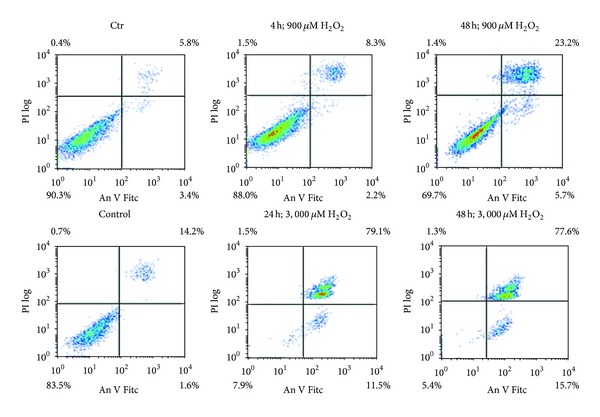
Dot plots of FITC-annexin V/PI flow cytometry. hMESCs were subjected to 900 *μ*M or 3,000 *μ*M H_2_O_2_ for 1 h with following H_2_O_2_ replacement and cell cultivation under normal conditions. Apoptosis was detected at indicated time points. Control (Ctr): untreated cells.

H_2_O_2_ is well documented to cause apoptosis by a dose-dependent manner in various cell types. According to our data, at 200 *μ*M H_2_O_2_, no apoptosis was detected in the cell population up to day 21; therefore we tested here whether H_2_O_2_ at a high concentration causes apoptosis in hMESCs. In order to detect apoptosis, annexin V/PI staining was performed. As presented in Figure 5, upper panel, 900 *μ*M H_2_O_2_ reduced the number of viable cells from 90.3% to 69.7% in 48 h after treatment by increasing the number of AnV/PI+ cells. Remarkably, no significant changes in the number of early apoptotic cells (AnV+) compared with control were observed for 48 h. By contrast, H_2_O_2_ at higher concentration (3,000 *μ*M) caused apoptosis with no evidence of necrosis in a similar pattern but with much stronger effect (Figure 5, lower panel). Thus, the apoptotic levels in H_2_O_2_-treated hMESCs were regulated by a dose-dependent manner. According to our preliminary results, apoptosis was mediated by both caspase-8 and activated caspase-3; however, the exact mechanism of H_2_O_2_-induced apoptosis in hMESCs remains to be further elucidated.

**Figure 6:**
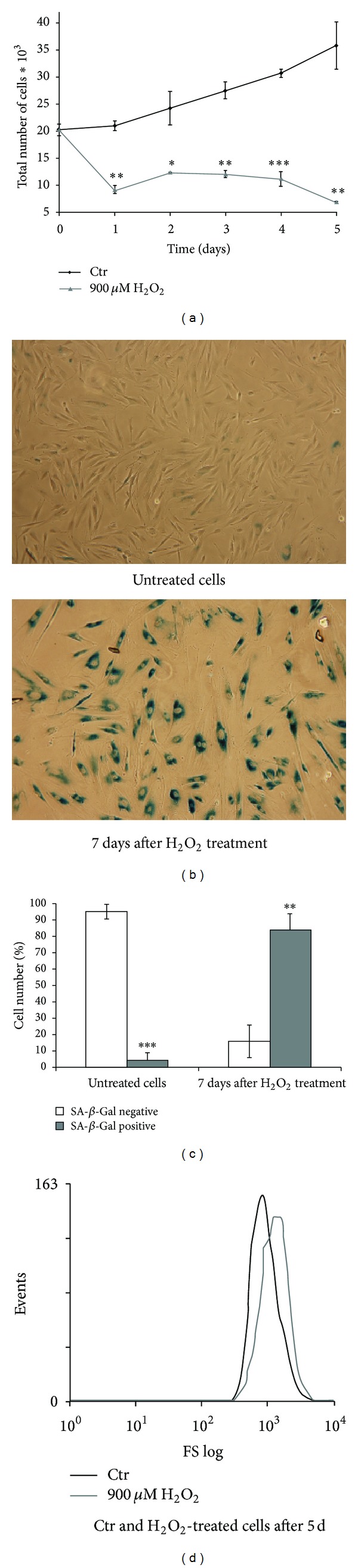
The senescent phenotype in hMESCs treated with 900 *μ*M H_2_O_2_. (a) H_2_O_2_-induced inhibition of cell proliferation (M ± SEM, *n* = 3). (b) SA-*β*-Gal staining. (c) Quantitative assay of SA-*β*-Gal-positive cells. (d) Cell hypertrophy detected by light-scattering cytometry. Significant difference was based on the Student's *t*-test (*P < 0.05, **P < 0.01, and ***P < 0.001). Control (Ctr): untreated cells.

Interestingly, 900 *μ*M H_2_O_2_ triggered not only delayed apoptosis but also led to the emergence of enlarged and flattened cells in the same cell cultures. Cell changes in the presence of both 200 *μ*M and 900 *μ*M H_2_O_2_ were similar in appearance. These observations prompted us to test if cell hypertrophy could be connected with premature senescence. As shown in Figure 6, 900 *μ*M H_2_O_2_ actually promoted the senescent morphology and SA-*β*-Gal staining, permanent growth arrest, and approximately 2-fold increase of cell size, pointing to premature senescence of the main part of cell population. Notably, the major senescence features induced by both 200 *μ*M and 900 *μ*M H_2_O_2_ were found to be alike. Together, these findings demonstrate that hMESCs exhibit high apoptosis resistance compared with human mesenchymal stem cells derived from both umbilical cord blood [9] and bone marrow [7], in which H_2_O_2_ above 200 *μ*M triggered apoptosis, whereas 100–150 *μ*M H_2_O_2_ induced senescence. Moreover, after exposure to sublethal doses of H_2_O_2_, senescent hMESCs acquired the increased stability in culture and displayed enhanced resistance to H_2_O_2_-induced apoptosis (data not shown). Many cell types acquire resistance to some apoptotic signals when they become senescent. So, senescent human fibroblasts resist apoptosis induced by oxidative stress or growth factor deprivation but do not resist Fas-mediated apoptosis [31, 32]. Resistance to apoptosis might in part explain the enhanced stability of senescent cells in culture. The mechanisms by which senescent cells resist apoptosis are poorly investigated. The senescence and apoptosis regulatory systems are supposed to communicate probably through their common regulator, p53 tumor suppressor protein [33]. 

In this study, we have provided the reliable evidence for our hypothesis that hMESCs are able to undergo the premature senescence in response to oxidative stress induced by H_2_O_2_ in a wide range of concentrations from 200 to 900 *μ*M. According to data obtained, entering senescence was accompanied with a rapid initiation of the cellular events, such as the changes of cell phenotype, the increase of SOD1, SOD2, and GPX1 expression, and upregulation of p21 without increase over time, leading to the irreversible cell cycle arrest and loss of proliferative potential. Since 2009, when phenomenon of stress-induced premature senescence in human mesenchymal stem cells was described for the first time, there were only a few publications concerning the oxidative stress-induced premature senescence of human mesenchymal cells derived from bone marrow [7, 8] and umbilical cord blood [9]. Even though both stem cell lines under sublethal stress respond with senescence, the major features of this process, in particular, dynamics of p21 accumulation and decline of cell proliferation rate, were extremely different, depending on the cell context. The precise molecular mechanism required to regulate the oxidative stress-induced premature senescence of human mesenchymal stem cells is far from understanding. Senescence program seems to develop in mesenchymal stem cells as a result of DNA damage response, leading to functional activation of either the p53/p21 or the p16INK4a (p16)/retinoblastoma protein (pRb) pathway, both of which can establish and maintain the growth arrest that is typical of senescence [23, 24]. The cyclin-dependent kinase inhibitors p16 and p21 may maintain pRb in active hypophosphorylated state [34, 35]. In turn, pRb halts cell proliferation by suppressing the activity of transcription factor E2F that regulates cell cycle progression. Our preliminary data, indicating a time- and dose-dependent formation of foci that contain phosphorylated histone H2AX (*γ*H2AX), activation of both ATM and p53, and upregulation of p21 expression, suggest that in hMESCs subjected to sublethal doses of H_2_O_2_ the senescence process may be controlled by the p53 pathway. In parallel, by monitoring the kinetics of p38 mitogen-activated protein kinase (MAPK) activation in H_2_O_2_-induced senescence of hMESCs, we have revealed a rapid and continued phosphorylation of p38 MAPK, indicating its possible role in the regulation of the premature senescence [36]. On the other hand, pRb was reported to induce growth arrest as a downstream molecule of p38 MAPK [37]. Taking into consideration these results, we cannot exclude the possibility that p16/pRb signaling cascade is also implicated in hMESCs senescence promotion.

Understanding the mechanisms of senescence process will be of great importance in developing applications of hMESCs in regenerative medicine to provide new strategies in autologous transplant and bioengineering. Primarily, hMESCs may be applied for cell therapy of infertility associated with decidualization insufficiency. Decidualization of endometrium is known to be an essential process for embryo implantation, placenta forming, and maintenance of pregnancy [38]. A noninvasive and easily available source for isolation of hMESCs, high proliferation activity during long-term cultivation, genetic stability, lack of tumorigenicity [39], and low immunogenicity make hMESCs a promising source of stem cells for clinical applications, including reproduction technology.

In summary, we have displayed for the first time that hMESCs in oxidative stress conditions undergo a premature senescence. Data obtained broaden a conception of mesenchymal stem cell senescence under oxidative stress. Taken together, the findings presented here and the data published allow us to assume that induction of premature senescence might be a common physiological response to sublethal oxidative stress in human mesenchymal stem cells of any origin.

## Abbreviations

hMESCs: Human endometrium-derived mesenchymal stem cells
HDF: Human diploid fibroblasts
SIPS: Stress-induced premature senescence
H_2_O_2_: Hydrogen peroxide
FACS: Flow-activated cell sorting
SA-*β*-Gal: Senescence-associated *β*-galactosidase
SOD1: Cytosolic superoxide dismutase
SOD2: Mitochondrial superoxide dismutase
GPX1: Glutathione peroxidase.
